# BZLF1 Governs CpG-Methylated Chromatin of Epstein-Barr Virus Reversing Epigenetic Repression

**DOI:** 10.1371/journal.ppat.1002902

**Published:** 2012-09-06

**Authors:** Anne Woellmer, Jose M. Arteaga-Salas, Wolfgang Hammerschmidt

**Affiliations:** 1 Department of Gene Vectors, Helmholtz Zentrum München, German Research Center for Environmental Health, Munich, Germany; 2 Adolf-Butenandt Institut, Ludwig-Maximilians-Universität, Munich, Germany; Emory University, United States of America

## Abstract

Epigenetic mechanisms are essential for the regulation of all genes in mammalian cells but transcriptional repression including DNA methylation are also major epigenetic mechanisms of defense inactivating potentially harmful pathogens. Epstein-Barr Virus (EBV), however, has evolved to take advantage of CpG methylated DNA to regulate its own biphasic life cycle. We show here that latent EBV DNA has an extreme composition of methylated CpG dinucleotides with a bimodal distribution of unmethylated or fully methylated DNA at active latent genes or completely repressed lytic promoters, respectively. We find this scenario confirmed in primary EBV-infected memory B cells *in vivo*. Extensive CpG methylation of EBV's DNA argues for a very restricted gene expression during latency. Above-average nucleosomal occupancy, repressive histone marks, and Polycomb-mediated epigenetic silencing further shield early lytic promoters from activation during latency. The very tight repression of viral lytic genes must be overcome when latent EBV enters its lytic phase and supports *de novo* virus synthesis in infected cells. The EBV-encoded and AP-1 related transcription factor BZLF1 overturns latency and initiates virus synthesis in latently infected cells. Paradoxically, BZLF1 preferentially binds to CpG-methylated motifs in key viral promoters for their activation. Upon BZLF1 binding, we find nucleosomes removed, Polycomb repression lost, and RNA polymerase II recruited to the activated early promoters promoting efficient lytic viral gene expression. Surprisingly, DNA methylation is maintained throughout this phase of viral reactivation and is no hindrance to active transcription of extensively CpG methylated viral genes as thought previously. Thus, we identify BZLF1 as a pioneer factor that reverses epigenetic silencing of viral DNA to allow escape from latency and report on a new paradigm of gene regulation.

## Author Summary

Latency is a fundamental molecular mechanism that is observed in many viruses. We reveal that the human herpes virus Epstein-Barr virus (EBV) uses cellular functions of epigenetic repression to establish latency in infected B cells and a previously unknown mechanism to escape from it. We show that the herpesviral DNA genome is transcriptionally silenced by cellular mechanisms during viral latency, which includes excessive methylation of EBV DNA *in vitro* and in its human host *in vivo*. Epigenetic modifications like high nucleosome density and repressive histone marks shield and inactivate lytic viral genes during latency. EBV's genuinely repressed chromatin poses the problem of efficient reactivation to support virus synthesis. *BZLF1* is the viral switch gene that induces the lytic phase of EBV's life cycle. We show here that this viral transcription factor erases static, repressive chromatin marks reversing epigenetic silencing. DNA methylation is preserved but no hindrance to lytic gene activation because BZLF1 directly binds to methylated viral DNA and overcomes heavily repressed chromatin without the need for active DNA demethylation. DNA demethylation has been thought to be a prerequisite for gene transcription but this virus falsifies this hypothesis and provides a new model for epigenetic gene regulation.

## Introduction

Activity and repression of eukaryotic genes correlate with the level of DNA methylation of promoter regions. Prominent models are ß-globin genes. Their sequential developmental activation and silencing in embryonic, fetal, and adult erythroid cells depends on the methylation status of DNA sequences near promoters of globin genes [1], [2 and references therein]. It appeared that CpG methylation is a stable epigenetic mark transmitting the repressed state of chromatin through mitosis to daughter cells. Little was known about dynamic demethylation (and methylation) events at promoters although demethylation is considered to be a prerequisite for gene activation at highly CpG-methylated promoter elements. It is now clear that gene activation can involve the rapid gain or loss of 5′-methylcytosine (5mC) residues in estrogen-responsive promoters [3], [4]. The methylation status of CpGs close to the transcription start site of the *pS2* promoter gene changes upon estrogen induction within minutes indicating that methylation of DNA is dynamic but also involves processes of reactive demethylation [5]. Erasure of DNA methylation and derepression of silenced chromatin has been observed in zygotes and primordial germ cells during fertilization and embryonic development, respectively. Recently, the responsible enzyme(s) were identified as members of the Tet (ten eleven translocation) family of proteins capable of catalyzing the conversion of 5mC to 5′-formylcytosine followed by the excision by thymine-DNA glycosylase and base excision repair [6]–[10]. Therefore, Tet proteins may drive the process of active CpG-demethylation, which is thought to be crucial to overcome transcriptionally repressed chromatin.

Epigenetic information like positioned nucleosomes or posttranslational modifications of N-terminal histone tails provides more flexibility to react to environmental cues. In inducible promoters nucleosome positions change depending on the activation state of the gene [11 for a recent review]. N-terminal modifications of histone tails can be highly dynamic as a distinct epigenetic state can be enzymatically reverted by particular histone-modifying enzymes erasing the previous modifications [12 for a recent review].

Certain histone modifications are flexible in principle but can be stable and heritable through many cell generations. For example, Polycomb group (PcG) proteins are regulators that repress genes by keeping a transcriptionally inactive state, which is mediated by H3K27 trimethylation. The common view is that the Polycomb repressive complex 2 (PRC2) acts as the “writer” of the repressed state. It establishes H3K27 trimethylation with its histone methyltransferases EZH1 or EZH2. A second Polycomb repressive complex, PRC1, is regarded as the “reader” of the epigenetic state. It recognizes histone H3K27me3 and acts as a silencing complex by ubiquitinating histone H2A [13] or by chromatin compaction of defined nucleosome arrays [14] leading to stably repressed chromatin loci. During specific stages of embryogenesis and stem-cell differentiation certain members of the trithorax group of proteins can remove the methyl groups at lysine 27 of histone 3 to re-install transcriptionally active genes [15], [16].

The balance between epigenetic stability and flexibility underlies the adaption of EBV to its human host. In infected cells this herpesvirus can adopt two different states, which depend on the epigenetic regulation of its genes. Upon infection of primary human B cells, the virus does not promote *de novo* virus synthesis but establishes a latent phase, characterized by the expression of a small set of viral genes, which promote cellular proliferation and contribute to viral oncogenesis [17]. Extensive DNA methylation of viral DNA [18], [19] is thought to contribute to overall gene silencing [20] whereas histone modifications such as H3K4me3 mark the few active promoters of latent genes together with the chromatin insulator protein CTCF and the viral factor EBNA1 [21], [22]. EBV's *EBNA* genes can be alternatively expressed from the latent viral *Cp*-, *Wp*- or *Qp*-promoters. Their states of DNA methylation were studied *in vivo* by several groups [23], [24] and *in vitro* in cell lines of different origins [25], [26]. They found that the methylation status of these promoters determines the expression profile of latent viral genes. In contrast to latent viral promoters, little is known about epigenetic marks at lytic promoters during latency when epigenetic silencing of most viral genes might guarantee the co-existence of EBV with its cellular host in the absence of viral *de novo* synthesis.

The eventual switch to EBV's lytic phase in latently infected cells is initiated by the expression of the viral *BZLF1* gene encoding the transcription factor BZLF1 (also called EB1, ZEBRA, Z, or Zta) [27], [28]. BZLF1 binds sequence-specifically to one class of DNA motifs, termed ZREs, but prefers a second class that contains methylated 5′-cytosine residues (5mC), termed meZREs [18], [29]–[31]. meZREs predominate in so-called early viral promoters [32]. Paradoxically, CpG methylation of meZREs is instrumental for the expression of essential lytic genes [33] and indispensable for virus synthesis [18], [32], changing the conventional view of DNA methylation solely as a repressive epigenetic feature.

EBV's closest relative, Kaposi's sarcoma-associated herpes virus (KSHV), relies on bivalent chromatin in its latent phase to reactivate the lytic phase of its life cycle [34], [35]. In contrast to this option, we report here on a novel mechanism that deliberately relies on methylated EBV DNA to promote induced transcription of a distinct class of viral genes. Our data suggests that, in combination with a very high concentration of highly methylated CpG dinucleotides, Polycomb-group (PcG) proteins introduce repressive modification of histones, which form densely arranged nucleosomes in order to shield the binding sites of BZLF1 in certain promoters of Epstein-Barr virus (EBV) to maintain latency. Upon lytic reactivation BZLF1 is expressed and gains access to compacted, highly repressive chromatin, where it binds to its CpG-methylated DNA motifs. BZLF1 binding induces nucleosomal eviction at BZLF1's cognate sites, erases repressive histone marks, recruits RNA polymerase II, and activates transcription of viral genes to trigger escape from latency. Surprisingly, CpG methylation is invariably maintained during this early lytic phase of EBV's life cycle and no barrier to active transcription. These attributes provide a novel paradigm for gene regulation in metazoan cells, which depend on stable chromatin repression to maintain their differentiation state and cellular identity but require the plasticity needed to respond immediately to developmental and environmental cues. EBV exploits these mechanisms in order to sustain its lifestyle.

## Results

### Early lytic BZLF1-regulated promoters encompass dense clusters of highly methylated CpG dinucleotides

**Figure 1:**
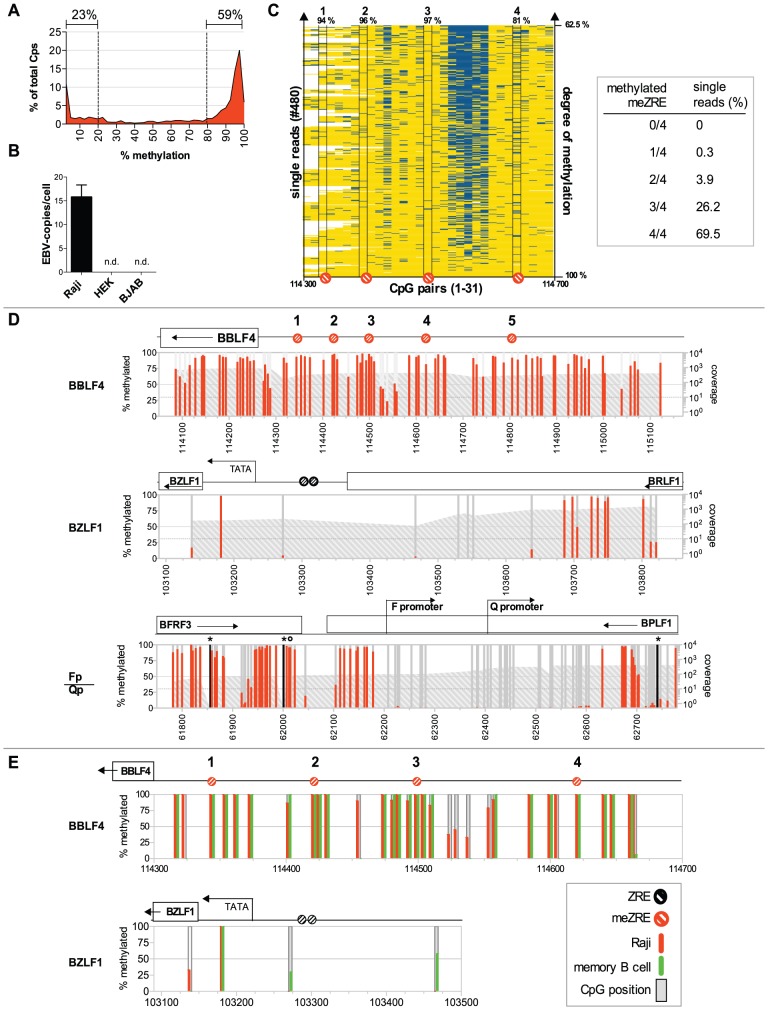
DNA methylation of EBV genomes in Raji cells and *ex vivo* isolated memory B cells. (**A**) Distribution of DNA methylation in Raji cells. (**B**) Determination of the number of EBV episomes in Raji cells. The absolute copy number of EBV DNA per cellular genome equivalent was determined by quantitative real-time PCR analysis of the *BZLF1* locus of EBV and compared with the *cytochrome c* locus as a cellular standard. Raji cells were determined to contain 16 copies of EBV per cell; HEK293 (HEK) and BJAB cells were used as negative controls. (**C**) Left panel: The 480 single reads of the deep bisulfite sequencing analysis of the *BBLF4* promoter were linearly ordered according to their average degree of CpG methylation. Reads with a high percentage of CpG methylation are at the bottom, reads with a lower percentage of CpG methylation are located at the top of the diagram. Yellow: methylated CpG, blue: unmethylated CpG, white: missing. The four identified meZREs within the *BBLF4* promoter are depicted below the diagram with their percentage of methylation shown above. Right panel: The single reads that contained the complete information of all four meZRE sites were analyzed for the co-occurrence of unmethylated meZREs on the same DNA molecule. 69.5% of all reads carried methylated CpGs in all four meZREs (69.5%). There was not a single read, in which all the meZREs were unmethylated. (**D**) DNA methylation of the lytic promoters *BBLF4* and *BZLF1* and the latent *Qp* promoter of EBV. Each bar represents a CpG dinucleotide plotted versus its B95.8 genome coordinate. The percentage of methylation is displayed on the left y-axis and indicated in red. The right logarithmic y-axis provides the sequencing coverage depicted as a grey area. Deep bisulfite sequencing detected single sequence variations between Raji DNA and B95.8 wildtype sequence. CpG dinucleotides missing in the Raji genome but present in B95.8 are indicated as black bars with a star on top, while additional CpG dinucleotides in Raji cells are indicated with a circle on top of the red or grey bar. Selected annotation of the EBV genome can be found above the graphs, with ZREs and meZREs indicated as black or red circles, respectively. The *BBLF4* promoter is rich in methylated CpG dinucleotides including all meZREs with high affinity for BZLF1. The *BZLF1* and the *Qp* promoters are mostly unmethylated. (**E**) Comparison of CpG DNA methylation in Raji cells and memory B cells of one healthy donor at two loci of the EBV genome. The percentage of CpG methylation in Raji cells and *ex vivo* purified memory B cells is depicted in red and green bars, respectively. PCR fragments were directly sequenced by the Sanger method and the resulting chromatograms were analyzed for the ratio of the height of the cytosine peak to the thymine peak, which reflects the rate of conversion after chemical bisulfite modification.

In the EBV genome a number of meZREs were identified, which presumably contain methylated CpGs [32]. We examined selected regions of the latent EBV genome with deep bisulfite sequencing to assess the state of cytosine methylation in the cell line Raji, which is our prototype of a B cell latently infected with EBV. After bisulfite modification genomic DNA from Raji cells was amplified by PCR with EBV-specific primer pairs. The analysis encompassed 26 regions of EBV with latent, early lytic, and late lytic gene promoters covering 27,869 bp of EBV's genome (Fig. S1A). The PCR products were pooled, sequenced, and the percentage of 5mCs was assessed [36]. The coverage of each CpG dinucleotide was 840 reads on average. A list with all CpGs analyzed is available upon request, a graphical view of selected promoters is shown in Fig. 1D and Fig. S1B.

Cytosines within CpG dinucleotides were methylated to 66% on average in Raji EBV DNA. We analyzed the distribution of EBV's DNA methylation in this latently infected B cell line. The analysis revealed that the methylation state of EBV DNA is bimodal with 23% hypomethylated (<20% methylation) and 59% hypermethylated CpGs (>80% methylation; Fig. 1A). Interestingly, the distribution peaks at the very left (<2.5% methylation) and the very right (>97.5% methylation) as shown in Fig. 1A, indicating that many viral CpG dinucleotides are either completely methylated or unmethylated.

The CpG-rich *BBLF4* promoter is a model of a BZLF1-regulated early lytic promoter [32] and its methylation state in Raji cells is shown in Fig. 1D. As predicted [32], the majority of CpG dinucleotides in this promoter carries 5mCs, which include the previously identified meZRE motifs. Our laboratory strain of Raji cells contains 16 copies of EBV genomes per cellular genome equivalent as determined by quantitative realtime PCR (Fig. 1B) suggesting that cytosine methylation in the *BBLF4* promoter is prevalent in all copies of EBV genomes present in Raji cells.

Our data so far did not exclude that unmethylated copies of EBV genomes might exist that could bind BZLF1 and e.g. exclusively support transcriptional reactivation of the *BBLF4* promoter upon induction of EBV's lytic phase (see below). To address this point we arranged single reads of the deep sequencing analysis of the *BBLF4* promoter according to their average degree of CpG methylation (Fig. 1C). We observed that (i) the majority of all 31 CpG dinucleotides present in the *BBLF4* promoter carries 5mC residues and not a single DNA molecule is entirely unmethylated, (ii) CpG methylation is variable at two hotspots while (iii) unmethylated meZREs are infrequent and rarely cooccur on the same DNA molecule. Our analysis excludes the existence of EBV genomes that escape CpG methylation in this model (Fig. 1C) but highlights the need to override CpG methylation and reverse epigenetic repression upon induction of EBV's lytic phase.

The *BBLF4* promoter is no exception as other early lytic promoters showed an equally high and homogeneous methylation at meZREs with a median methylation rate of 95.3% (Fig. S1B and Table S1). In contrast, active latent viral genes and their promoters like the *Qp* promoter (Fig. 1D, lower panel) were virtually free of methylated CpG dinucleotides in Raji cells resembling cellular CpG islands with a very high density of CpGs spared from DNA methylation.

Next, we wanted to assess the methylation profile of EBV DNA in cells, which are the latent reservoir of EBV in its human host. We sorted memory B cells from PBMCs of healthy donors by multiparameter FACS enriching CD19 positive, CD38 negative, IgD negative, and CD27 positive cells. Cellular DNA was extracted and parts of EBV's genome were analyzed by conventional bisulfite sequencing. The frequency of EBV-infected memory B cells was reported to be very low with only one EBV-positive cell out of 10^4^–10^6^ cells [37], but we succeeded in obtaining information of the *BBLF4* and *BZLF1* promoters from DNA samples of two healthy individuals. The *BBLF4* promoter was highly CpG-methylated *in vivo* (Fig. 1E) but the pattern was not entirely identical to that of Raji DNA. The four CpGs, which constitute the meZREs of *BBLF4*, were fully methylated in memory B cells *in vivo*. In contrast, CpG dinucleotides in the *BZLF1* promoter are rare and were unmethylated in Raji DNA and weakly or variably methylated in memory B cells (Fig. 1E) indicating that this locus is probably not controlled by CpG methylation *in vivo* or *in vitro*.

### Nucleosomal occupancy in EBV DNA

In addition to cytosine methylation, the density and the position of nucleosomes might also contribute to the transcriptionally repressed state of EBV DNA during latent infection. We therefore investigated the nucleosomal occupancy of EBV's genomic DNA with a particular focus on BZLF1-regulated promoters in Raji cells. This cell line also provides a model for viral reactivation because ectopic expression of *BZLF1* efficiently induces EBV's lytic phase in Raji cells (see below). Lytic induction does not lead to *de novo* synthesis of virus progeny because the EBV genome in Raji cells has a deletion of the *BALF2* gene abrogating viral DNA amplification in the lytic phase. As a consequence, the late lytic phase is blocked, which is advantageous for the exact, unequivocal analysis of viral DNA, viral chromatin, and transcripts of early lytic genes shortly after induction. We introduced a conditional allele of *BZLF1* into Raji cells to analyze possible changes of epigenetic modifications of viral chromatin at the onset of EBV's lytic phase. The coding sequences of *BZLF1* and *green fluorescent protein* (*GFP*) were placed under the control of the bidirectional conditional promoter (Fig. S2A). Addition of doxycycline led to about 90% GFP-positive cells (Fig. S2B), a rapid induction of *BZLF1* (Fig. S2C), and the expression of the BZLF1 target gene *BRLF1* in more than 50% of GFP positive cells (Fig. S2D).

In a first approach, chromatin of different Raji cell derivates was studied with an MND-on-Chip analysis (mono-nucleosomal DNA-on-Chip). MND-on-Chip experiments rely on the different accessibility of DNA to Micrococcal Nuclease (MNase) cleavage in the context of chromatin. Digestion of chromatin with MNase leads to the degradation of free DNA, whereas nucleosomal DNA is protected and can be isolated and identified in e.g. microarray hybridization.

Mononucleosomal DNA and sonicated input DNA were labeled with fluorochromes and hybridized to a high-resolution custom-made EBV microarray. The experiments were performed with three different cell lines. Parental Raji cells were analyzed as a model of the latent state. Chromatin of lytically induced and sorted Raji-BZLF1 cells addressed nucleosomal occupancy during EBV's lytic phase. Raji-BZLF1ΔTAD cells constitutively expressed a truncated BZLF1 protein lacking its N-terminal transactivation domain [32] to study the effect of BZLF1's DNA binding domain (DBD) on nucleosomal occupancy at BZLF1-regulated promoters.

**Figure 2:**
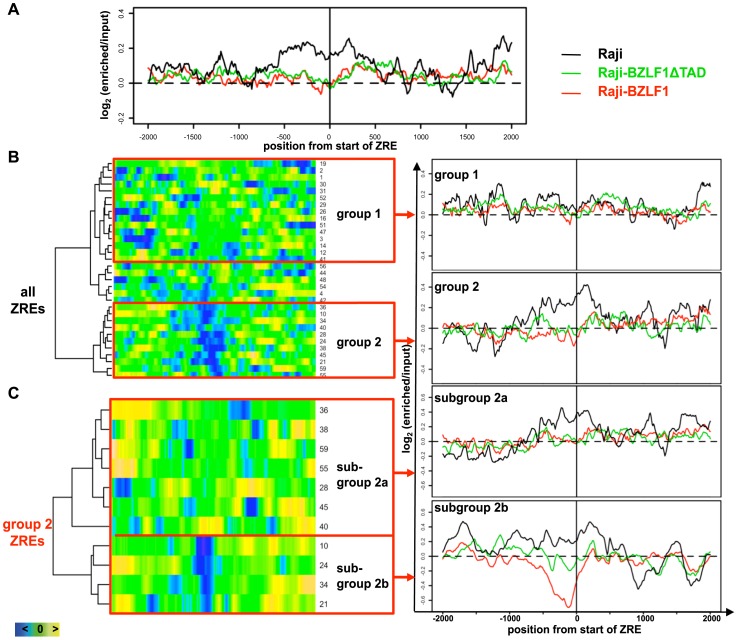
Nucleosome occupancy in BZLF1-regulated promoters. (**A**) Overlay of the average nucleosome occupancy profiles of 32 ZREs (Table S2) in parental Raji cells (black line) and two derivatives. Raji-BZLF1ΔTAD cells (green line) constitutively express a truncated BZLF1 version without its transactivation domain. Raji-BZLF1 cells (red line) express wild-type BZLF1 protein 15 hours after doxycycline induction. The nucleosome occupancy was analyzed in a window of ±2000 bp, centered at the start position of each ZRE. (**B**) Comparison of the nucleosome occupancy of latent Raji and lytically induced Raji-BZLF1 cells. Left panel: a heat map of 32 single ZREs (Table S2) shows the subtractions of the log2-ratios of lytically induced Raji-BZLF1 cells and latent Raji cells. A dendrogram of the heat map matrix shown on the left side indicated two most divergent groups, group 1 and 2. Right panel: The overlay of the average nucleosomal occupancy for group 1 ZREs and group 2 ZREs were obtained as in (A). (**C**) Comparison of the nucleosome occupancy of Raji-BZLF1ΔTAD and lytically induced Raji-BZLF1 cells. A second heat map and dendrogram comprises subtractions of the log2-ratios of lytically induced Raji-BZLF1 cells (full length BZLF1) and Raji-BZLF1ΔTAD cells (truncated BZLF1) of the eleven single ZREs of group 2. Subgroup 2a ZREs comprised ZREs of the upper and lower cluster of the dendrogram on the left (see Table 1 for their grouping).

32 ZREs were selected, which fulfilled certain criteria to ensure their being informative (Table S2). The “enriched versus input” log_2_-ratios indicated the occupancy of DNA with nucleosomes. The log_2_-ratios of the selected ZREs were averaged and the average nucleosome occupancy profiles of the three Raji derivates were overlaid in a window ±2000 bp, centered at the start of the ZRE (Fig. 2A). The average nucleosome occupancy profile displayed increased nucleosome occupancy at ZREs during latency (parental Raji cells, black line), indicated by a high log_2_-ratio. The elevated log_2_-ratio dropped after lytic induction (Raji-BZLF1 cells, red line) and after binding of truncated BZLF1 (Raji-BZLF1ΔTAD cells, green line). This observation indicated that binding of full length BZLF1 but also the C-terminal half of BZLF1 with its DBD caused a general loss of nucleosomes at ZREs.

The log_2_-ratios of the 32 individual ZREs in lytically induced Raji-BZLF1 cells and parental Raji cells were subtracted and ranked in a heat map shown in the left panel of Fig. 2B. A cluster analysis was performed to probe for possible functional groups among all ZREs. The resulting dendrogram shown on the left side of the heat map identified three functional groups of ZREs. For the subsequent analysis the two most divergent groups, group 1 and group 2 were selected (Fig. 2B). The average nucleosome occupancy profile of group 1 ZREs in parental Raji cells during latency (Fig. 2B, right panel, black line) did not display elevated log_2_-ratios at the ZRE sites in contrast to group 2 ZREs. Their average log_2_-ratios dropped after induction of the lytic phase (red line), and constitutive binding of BZLF1 caused a similar reduction of average nucleosome occupancies at group 2 ZREs (green line).

Next, we analyzed the differences between Raji-BZLF1ΔTAD cells that express the truncated BZLF1 protein constitutively and lytically induced Raji-BZLF1 cells. This analysis addressed the role of BZLF1's N-terminal transactivation domain on nucleosomal displacement. Subtraction of the log_2_-ratios of the eleven ZREs of group 2 in lytically induced Raji-BZLF1 cells and Raji-BZLF1ΔTAD cells are visualized in a second heat map (Fig. 2C). A cluster analysis and the resulting dendrogram distinguished two subgroups. Subgroup 2a showed similar average nucleosome occupancies between the two datasets, indicating a similar displacement of nucleosomes, as compared to parental Raji cells (Fig. 2C, upper right panel). Average nucleosome occupancy profiles of subgroup 2b showed high nucleosome occupancy in latent parental Raji cells (black line) as expected. Binding of truncated BZLF1 (green line) caused a small drop in the log_2_-ratios, suggesting lower nucleosome occupancy on average. The induction of the lytic phase in Raji-BZLF1 cells resulted in a collapse of the log_2_-ratios (red line), indicating the formation of hypersensitive sites. Control experiments with 32 randomly chosen positions of the EBV genome lacking any ZRE did not reveal differences between the datasets (Fig. S3).

Our findings implied a functional classification of BZLF1-regulated genes. Seven genes are essential for EBV's DNA replication in the lytic phase: *BMLF1*, *BRLF1*, *BMRF1*, *BBLF2*/3, *BBLF4*, *BALF2*, and *BALF5* [38], [39] (*BALF2* is deleted in Raji EBV DNA and excluded from this analysis). Five of the remaining six promoters of these genes make up subgroup 2b or belong to subgroup 2a, indicating that theses promoters are repressed very tightly during latency. The promoters of *BMLF1*, *BRLF1*, and *BMRF1* (subgroup 2b) probably require the local binding of wild-type BZLF1 for their chromatin remodeling upon lytic induction. The promoters of *BBLF2/3* and *BBLF4* (subgroup 2a) also appeared tightly repressed during latency, but binding of truncated BZLF1 was sufficient to rearrange the promoter nucleosomes. The seventh gene important for lytic replication, DNA polymerase *BALF5*, contains a single ZRE in its promoter [32], which did not fall into any analyzed group. Promoters of group 1 are not regulated by BZLF1 at the level of nucleosomal rearrangements. None of them belongs to the seven genes, which are required for EBV's lytic DNA replication. Table 1 lists the ZREs hierarchically and according to their functional groups, Fig. S4 provides the graphical representation of examples of two members of each group.

**Table 1:** Catalog of functional groups of ZREs.

group	ID	location
subgroup 2b	10	*oriLyt*
	24	*BMLF1* promoter
	34	*BRLF1* promoter
	21	*BMRF1* promoter
subgroup 2a	40	*BBLF2*/3 promoter
	38	*BBLF4* promoter
	55	*BALF5* reading frame
	36	*BKRF3* promoter
	28	*BLLF1* reading frame
	45	*BDLF3* promoter
	59	*BNLF2a*/b promoter
group 1	19	*Barf1* promoter
	2	*BNRF1* reading frame
	1	*BNRF1* reading frame
	30	*EBNA3B* reading frame
	31	*BZLF1* promoter
	52	*BILF2* promoter
	29	n.o.s.*
	26	*BSRF1* promoter
	16	*BPLF1* reading frame
	51	*BdRF1* promoter
	47	*BcLF1* reading frame
	3	*BCRF1* reading frame
	14	*BFLF1* promoter
	12	*BFLF2* promoter
	41	*BGLF5* promoter

* n.o.s. not otherwise specified.

### Validation of MND-on-Chip analysis

MND-on-Chip experiments indicated a loss of nucleosomes at ZREs, which was validated in two additional, independent experiments with parental Raji cells (latent) and doxycycline induced Raji-BZLF1 cells (lytic).

**Figure 3:**
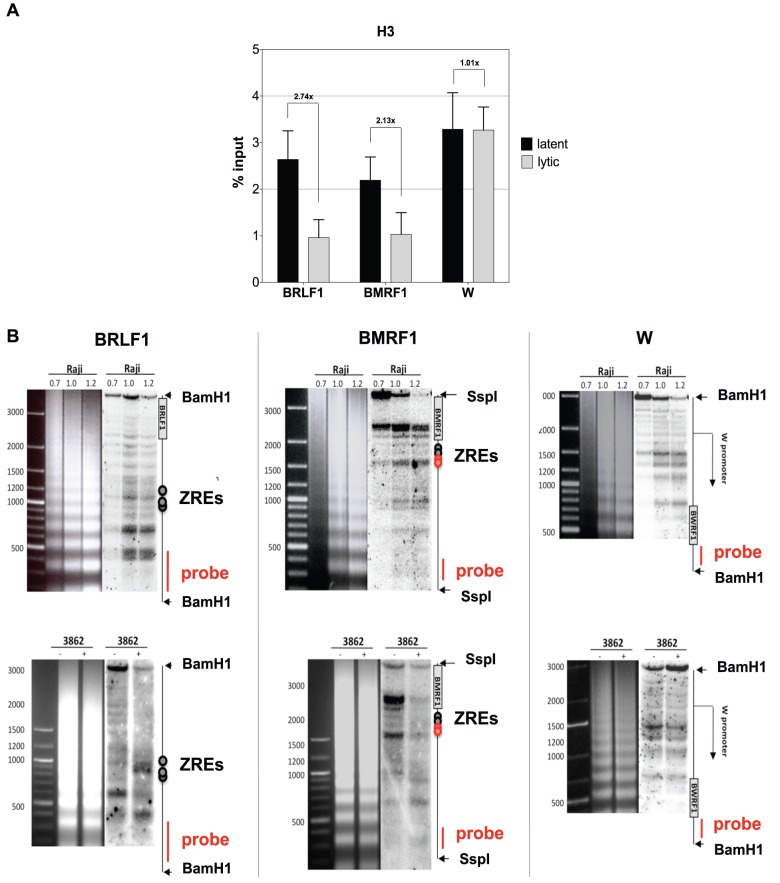
Chromatin Immunoprecipitation and indirect endlabeling experiments confirm the loss of nucleosomes at ZREs. (**A**) Chromatin Immunoprecipitation (ChIP) of histone H3. Histone H3-ChIPs confirmed the loss of nucleosomes in two promoters containing ZREs (*BRLF1* and *BMRF1*) in lytically induced cells. In contrast, the ZRE-free latent *W* promoter was not affected. The results of three independent experiments were averaged. Data are represented as mean +/− SD. (**B**) Indirect endlabeling experiments confirmed the microarray hybridization and ChIP results. Upper panels: Indirect endlabeling experiments in Raji cells are shown covering three different promoters. Lower panels: The three regions were re-analyzed in uninduced Raji-BZLF1 cells (−) or after induction with 100 ng/ml doxycycline overnight (+). The patterns in uninduced cells were similar to the ones seen in parental Raji cells. Induction of the lytic phase led to rearrangements in the nucleosomal pattern at the ZRE-containing *BRLF1* promoter and the *BMRF1* promoter but not in the *W* promoter, which lacks ZREs.

Chromatin immunoprecipitations with a histone H3-specific antibody resulted in the enrichment of nucleosomal DNA containing histone H3 at two different promoters that contained ZREs (*BRLF1* and *BMRF1*) in comparison to the ZRE-free *W* promoter (*Wp*) during latency in parental Raji cells (Fig. 3A; 2–3% of input DNA). The ZRE-containing promoters of *BRLF1* and *BMRF1* showed a clear reduction of the histone H3 signal (1% of input DNA) in lytically induced Raji-BZLF1 cells but the ZRE-free *Wp* was not affected by lytic induction (Fig. 3A).

These three promoters were re-analyzed in indirect endlabeling experiments. Chromatin was treated with limited amounts of MNase and cleaved with appropriate restriction endonucleases at sites close to the regions of interest. DNA was purified, separated on agarose gels, and transferred to nylon membranes by Southern blotting. The membranes were hybridized to radioactively labeled probes that were complementary to regions downstream and close to the cleavage sites of the restriction endonucleases. The bands that are visible in the autoradiograms show the boundaries of nucleosomes and hypersensitive sites. The migration of the bands indicates the distance of the boundaries from the restriction endonuclease cleavage sites in the individual loci as shown graphically on the right side of the autoradiograms in Fig. 3B.

Top panels in Figure 3B show the results in parental Raji cells. Raji chromatin was partially digested with increasing amounts of MNase as indicated and analyzed for nucleosome occupancies in the promoter regions of *BRLF1*, *BMRF1*, and *Wp*. The restriction endonuclease cleavage sites, the probe location, and selected features of EBV's genome are depicted on the right side of the autoradiograms.

The lower panels in Figure 3B show the same promoter sites in Raji-BZLF1 cells, without or after addition of doxycycline and overnight induction of the lytic phase of EBV. The uninduced cells displayed a pattern similar to the latent parental Raji cells (Fig. 3B, upper panels) as expected. The induced expression of *BZLF1* caused major rearrangements in the promoters of *BRLF1* and *BMRF1*, but not in the ZRE-free *W* promoter. The *BRLF1* promoter acquired a pattern that was typical for a hypersensitive site, which is situated in the same location as the *BRLF1* ZRE sites, confirming the microarray and ChIP experiments. The *BMRF1* promoter showed overall reduced signal intensities in bands that were more than 1000 bp apart from the restriction endonuclease cleavage site after lytic induction, indicating a loss of nucleosomes in this BZLF1-regulated promoter as well.

### CpG methylation of EBV DNA is preserved in B cells supporting lytic viral transcription

**Figure 4:**
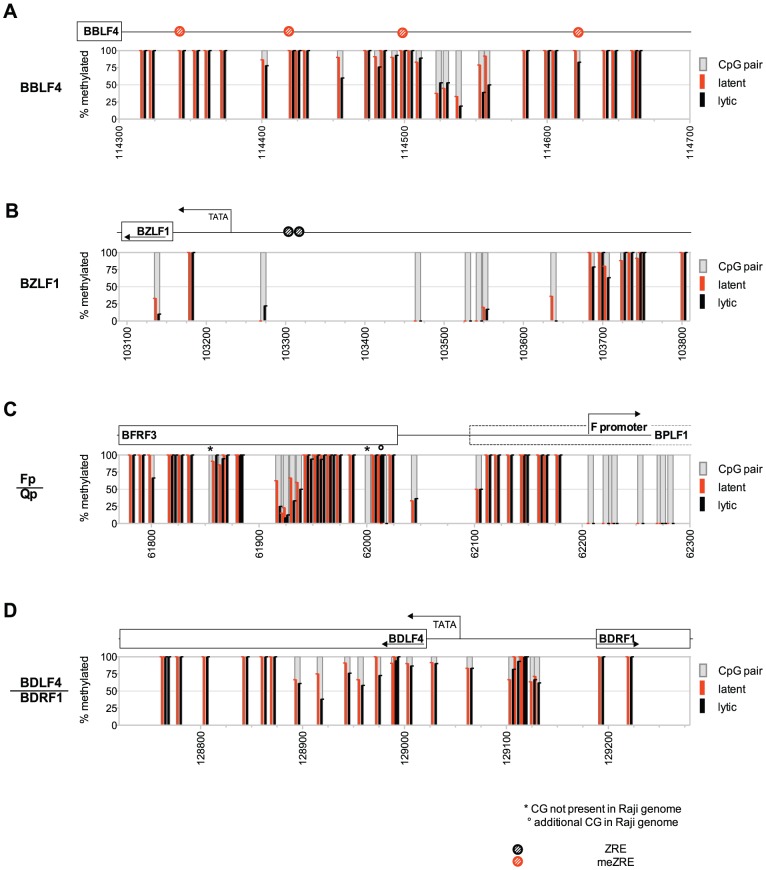
The DNA methylation profile of Raji EBV DNA does not change upon induction of EBV's lytic phase. DNA of Raji cells or Raji-BZLF1 cells, which had been induced with doxycycline for 15 hours and sorted for expression of GFP, were treated with bisulfite and amplified with primers specific for (**A**) the early lytic *BBLF4* promoter, (**B**) the immediate early *BZLF1* promoter, (**C**) the latent *Qp/Fp* promoter, and (**D**) the late lytic *BDLF4*/*BDRF1* promoter. PCR fragments were directly sequenced by the Sanger method as in Fig. 1E. The percentage of CpG methylation in Raji cells (latent) is depicted in red; the percentage of methylation in lytically induced Raji-BZLF1 cells (lytic) is indicated as black bars.

We considered that the induced expression of *BZLF1* and subsequent eviction of nucleosomes lead to an active demethylation of EBV DNA fostering chromatin reactivation and transcriptional activation. The methylation state of four selected regions of the viral genome covering the latent *Fp/Qp* promoter and three lytic promoters representing an immediate early, an early, and a late gene were determined after induction of the lytic cycle (Fig. 4). Raji cells with the conditional *BZLF1* allele (‘Raji-BZLF1’) were induced with doxycycline for 15 hours and sorted for GFP-positive cells to obtain a pure population of lytically induced cells. Cellular DNAs of lytically induced Raji-BZLF1 and parental Raji cells were isolated, bisulfite-treated, amplified with suitable primers spanning the *Fp/Qp* promoter, the *BZLF1* promoter, the *BBLF4* promoter, and the *BDRF1* promoter, and directly sequenced. There was no discernable difference between the DNA methylation pattern of parental Raji cells and lytically induced Raji-BZLF1 cells at any locus (Fig. 4) indicating that active demethylation of the viral genome is not part of EBV's lytic phase.

We wanted to challenge the possibility that BZLF1 might induce transcription from partially unmethylated templates of EBV DNA. BZLF1 could bind to unmethylated meZRE-sites, which, nevertheless, are infrequent in Raji DNA during latency (see above, Fig. 1) and only weakly bound by BZLF1 [32]. Towards this end we employed a BZLF1-specific antibody and performed chromatin-immunoprecipitation experiments followed by direct bisulfite sequencing termed ChIP-BS-seq (Fig. S5). If BZLF1 bound to non-methylated meZREs and/or preferentially supported transcription from partially methylated templates, we would expect an enrichment of DNA with a reduced frequency of methylated CpGs as compared to viral DNA present in latently infected Raji cells. Our results demonstrated that BZLF1-bound DNA was indistinguishable from EBV DNA prevalent in Raji cells during latency (Fig. S5). This finding further supported our working hypothesis that (i) lytic reactivation relies on methylated meZREs and (ii) BZLF1-controlled transcription originates from methylated DNA templates.

### Histone modifications and chromatin-modifying enzymes in EBV DNA

**Figure 5:**
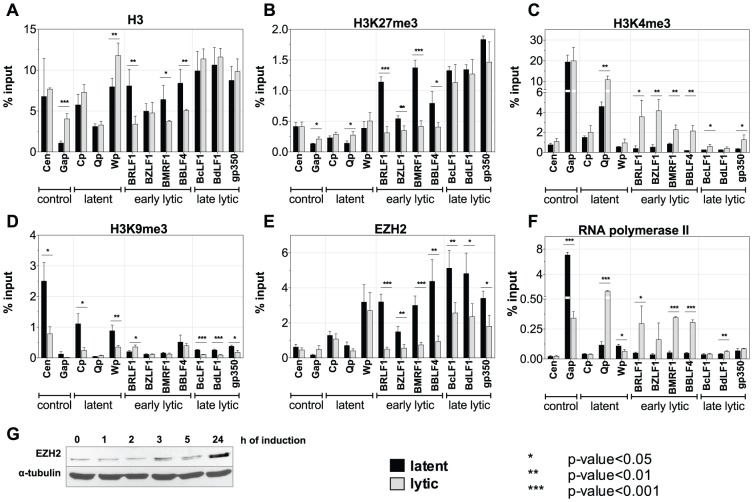
Chromatin state of Raji cells in the latent and the lytic phase. Three independent sets of chromatin immunoprecipitations (ChIPs) followed by quantitative PCR analysis of latent, early lytic, late lytic viral promoters, and cellular control loci are shown. Black bars represent uninduced, latent Raji-BZLF1 cells; grey bars represent lytically induced cells after treatment with doxycycline for 15 h. Data are represented as mean +/− SD. Asterisks indicate the p-value of each data point (*** p-value<0.001, ** p-value<0.01, * p-value<0.05). (**A**) histone H3; (**B**) repressive H3K27me3 histone modification; (**C**) activating H3K4me3 histone modification; (**D**) repressive H3K9me3 histone modification; (**E**) histone methyltransferase EZH2; (**F**) PolII ; (**G**) Western blot immunodetection of EZH2.

We analyzed promoters representing all different classes of EBV genes and included cellular control loci with chromatin immunoprecipitations (ChIPs) using antibodies against various histone modifications or certain chromatin-associated proteins (Fig. 5). panH3-specific ChIP experiments confirmed the results of the microarray analysis: early lytic promoters and late lytic promoters were enriched in histone H3 during latency but induction of the lytic phase caused a selective loss of histone H3 at early lytic promoters, only (Fig. 5A). The *BZLF1* promoter is an exception; lytic induction does not lead to a displacement of histone H3, which is in accordance with our microarray analysis (Table 1).

We also determined whether lytic promoters carry specific chromatin marks during latency. ChIP experiments with an anti-H3K9me3 antibody indicated that this repressive mark is present at some EBV-loci but that it is not important for the regulation of lytic promoters, as induction of the lytic phase did not alter their occupancy with this modification (Fig. 5D). In sharp contrast, ChIP experiments with an anti-H3K27me3 antibody revealed that all lytic promoters are characterized by this histone modification, which presumably is involved in their efficient repression during latency (Fig. 5B). Induction of the lytic phase selectively erased or reduced this modification at early lytic promoters, consistent with their reactivation.

High levels of H3K27me3 are a hallmark of Polycomb repression; therefore we assessed the occupancy of EBV promoters with the H3K27me3 methyltransferase EZH2, which is a protein component of the Polycomb repressive complex 2, PRC2. ChIPs with an EZH2-specific antibody perfectly matched the results of H3K27me3 suggesting that this methyltransferase is responsible for trimethylation of H3K27 in repressed EBV promoters (Fig. 5E). Interestingly, late lytic promoters, which do not support transcription in this model also showed a reduction in EZH2 levels, but to a lower extent.

The loss of EZH2 at EBV promoters did not result from a reduction of cellular EZH2 steady-state levels after the induction of EBV's lytic phase (Fig. 5G) but was a locus-specific phenomenon. These results demonstrated that (i) Polycomb repression is important to maintain EBV's lytic genes in a silent state during latency, and (ii) induction of EBV's lytic cycle eliminates this mark relieving the tight repression.

We also wanted to assess the mode of activation of early lytic promoters on the chromatin level. In Fig. 5C, the activation mark H3K4me3 appeared enriched at the transcriptionally active *Q* promoter (*Qp*) during latency, but H3K4me3 was undetectable at early lytic and late lytic promoters. Induction of the lytic phase increased the levels of H3K4me3 at *Qp* as well as early lytic promoters. Late lytic promoters also showed a minor enrichment of H3K4me3 marks upon lytic induction. ChIP experiments with an anti-PolII antibody showed no significant levels in the latent state (Fig. 5F) but in lytically induced cells, PolII was recruited to the latent *Q* promoter as well as early lytic promoters. In contrast, PolII was not detectable at late lytic promoters after lytic induction.

### Chromatin Remodeling in EBV is a temporally controlled event

**Figure 6:**
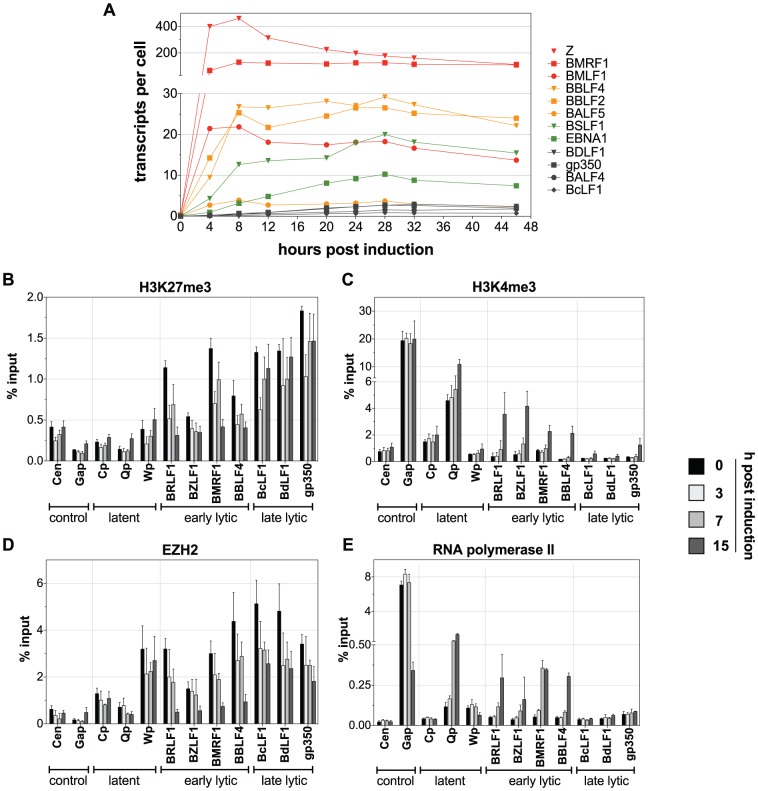
Kinetics of promoter activation after induction of EBV's lytic phase. (**A**) Evaluation of lytic transcript levels by quantitative RT-PCR. Raji-BZLF1 cells were induced with 100 ng/ml doxycycline for 46 h. A fraction of the cells was harvested every four hours. The temporal induction of different EBV genes suggests four different functional groups of viral genes. Group 1 (red) responds within four hours to the addition of doxycycline and encompasses *BZLF1 (Z)*, *BMRF1*, and *BMLF1*. Group 2 (orange) consists of *BBLF4*, *BBLF2*, and *BALF5*. They reached the expression peak eight hours post induction. *EBNA1* and *BSLF1* levels increased slowly over time (group 3, green) peaking at 28 hours after induction, while the late lytic genes (group 4, grey) showed no or only a very low expression upon lytic induction with kinetics comparable to group 3. (**B–E**) Chromatin immunoprecipitation experiments of Raji-BZLF1 cells upon lytic stimulation determined the occupancy of histones and their posttranslational modifications and the proteins EZH2 and RNA polymerase II in time course experiments. The experiments were conducted as described in Fig. 5. The loss of the repressive chromatin mark H3K27me3 and of the H3K27me3 methyltransferase EZH2 could already be detected after three hours post induction with doxycycline (B, D). The increase of the activation mark H3K4me3 was only visible 15 hours post induction (**C**) Binding of RNA polymerase II to early lytic promoters could be detected after 15 hours, but the very early responder BMRF1 was occupied by the protein already 7 hours post induction in line with the RT-PCR analysis, which identified this gene to be quickly induced upon lytic stimulation (**E**).

To address the kinetics of lytic gene activation quantitative RT-PCR analyses of Raji-BZLF1 cells documented the induction of PolII-mediated transcription of selected viral genes in a time course experiment (Fig. 6A). Raji-BZLF1 cells were induced with 100 ng/ml doxycycline for 46 h. RNA was prepared every four hours. Expression of a set of early and late lytic genes was tested together with the latent gene *EBNA1.* Absolute transcript levels were calculated on the basis of a single cell. The kinetics of induction differed among the early lytic genes indicating that some genes are direct targets of BZLF1, while other genes are probably induced by a combination of transcription factors or are secondary targets of BZLF1. The expression levels of the transgene *BZLF1* peaked after four hours of doxycycline induction. Expression of *BMRF1* and *BMLF1* was equally fast. The *BBLF4*, *BBLF2*, and *BALF5* genes were maximally expressed eight hours post induction. *BSLF1* and *EBNA1* levels continuously increased for a time period of 28 h of induction. Late lytic genes were not expressed or at very low levels, only.

We also followed the changes on EBV's chromatin over time to analyze the order of events contributing to the activation of early lytic promoters. ChIP experiments were conducted similar to the experiments described above with two additional, early time points after doxycycline induction of *BZLF1*. Polycomb repression of EBV promoters was substantially reduced as early as three hours post induction with signals decreasing further (Fig. 6B and D). Surprisingly, H3K27me3 initially also decreased at late lytic promoters three hours after BZLF1 induction but quickly recovered thereafter.

Compared to the rapid loss of repressive chromatin marks, activation marks and recruitment of PolII were slow processes. A significant increase in H3K4me3 signals became apparent 15 hours post induction similar to PolII (Fig. 6C and E). Only the *BMRF1* and the *Q* promoter showed a significant enrichment for PolII 7 hours after adding doxycycline followed by H3K4me3 modifications indicating that these promoters respond very early to lytic induction. As a consequence, *BMRF1* together with *BZLF1* and *BMLF1* was one of the first genes reaching high steady-state levels of transcripts (Fig. 6A). Our time course studies indicated a sequential order of events at viral chromatin: Polycomb repression is relieved before PolII is recruited to the promoter sites inducing viral gene expression.

**Figure 7:**
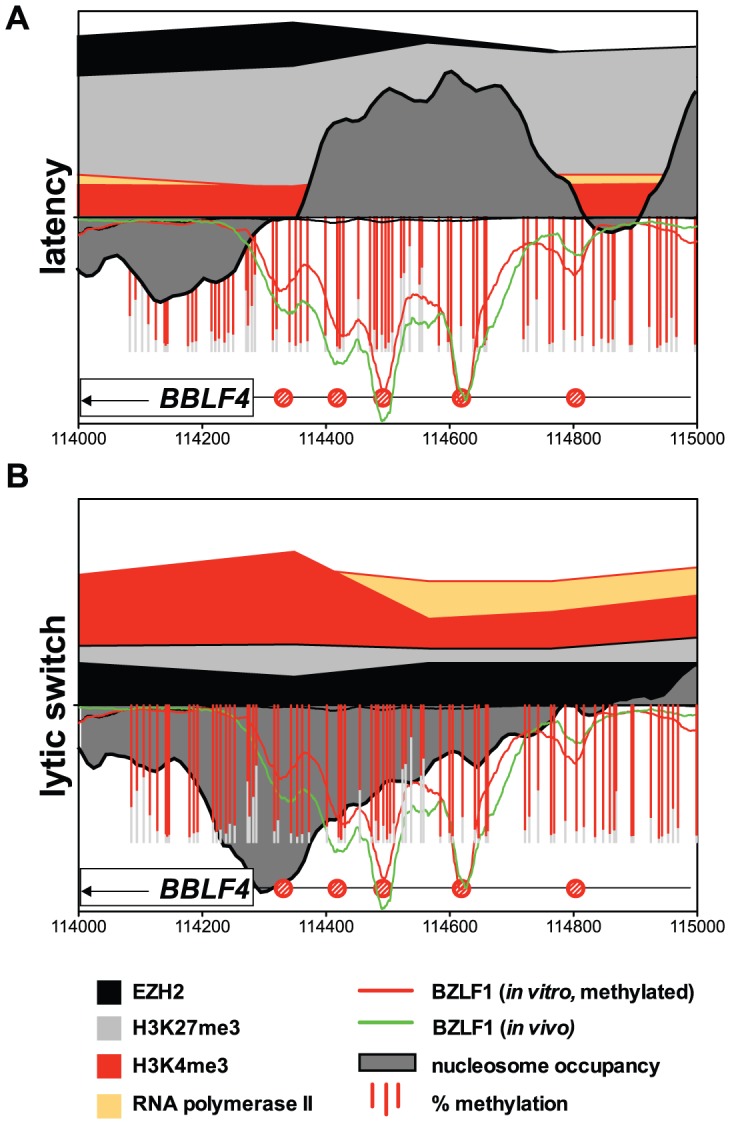
Epigenetic regulation of EBV: Close-up of the *BBLF4* promoter. On the lower half of the y-axis the binding profile of BZLF1 *in vivo* is shown as a green line, it's binding to methylated DNA *in vitro* as a red line. Red bars indicate the extent of CpG methylation (Fig. 1D). The dark grey area indicates relative nucleosomal occupancy. On the upper half of the y-axis, the black area indicates EZH2, the light grey area H3K27me3, the red area H3K4me3, and the orange area PolII. Results are provided as relative, dimensionless numbers; their scaling is identical in both graphs. (**A**) Repressive modifications characterize the *BBLF4* promoter during latency. (**B**) A hypersensitive site becomes established at meZREs immediately upstream of the coding sequence of *BBLF4* (grey area). H3K27me3 and EZH2 are lost and H3K4me3 and PolII indicate active transcription.

Figure 7 summarizes all epigenetic and functional data obtained in this study with the *BBLF4* promoter as a representative example. The promoter comprises several highly methylated meZREs [32]. During latency its nucleosomal occupancy was high and the complete region displayed high levels of H3K27me3 and EZH2. Activation marks and PolII were not detectable during latency. The situation changed dramatically upon lytic induction as the promoter was completely remodeled: nucleosomes and repressive modifications were evicted and erased, respectively. Instead, the H3K4me3 levels and PolII occupancy rose, inducing the transcription of *BBLF4*. It is worth noting that all these dramatic changes occur at chromatin encompassing densely arranged CpGs with highly methylated cytosine residues, which remain unaltered throughout the early lytic phase of EBV's life cycle.

## Discussion

Herpes viruses establish a life-long infection in their hosts. Success of infection relies on two principles: (i) in latently infected cells the promoters of lytic genes must be tightly repressed to evade immune recognition of their products by the infected host and (ii) induction of the lytic phase has to proceed rapidly and synchronously to escape from latency and virus-specific effector T cells. We found that repression and dynamic reactivation of viral genes are both governed by epigenetic mechanisms.

Repressed lytic promoters of EBV are associated with extensive DNA methylation, high nucleosome occupancy, and H3K27me3 histone marks, which are repressive modifications transmitted by Polycomb group (PcG) proteins leading to compaction of chromatin. Repressive H3K9me3 histone marks were detectable at low levels as reported recently [40], but very much in contrast to H3K27me3, H3K9me3 modifications are not removed upon viral reactivation suggesting that the low level of these modifications are not central to maintaining a repressed chromatin in EBV. The activation mark H3K4me3 and PolII were undetectable at lytic promoters during latency, indicating that EBV's latent DNA is not associated with bivalent chromatin in contrast to latently KSHV-infected cells.

DNA methylation is a prerequisite for the activation of EBV's early lytic promoters that rely on meZREs [18], [32]. Upon lytic reactivation, CpG-methylation of viral DNA was unaltered but repressive histone marks were erased and nucleosomes were evicted in a subset of ZREs, which led to a local opening of promoters and loading of the transcription machinery.

Only a subset of genes required the N-terminal transactivation domain of BZLF1 for an efficient nucleosome eviction, presumably by attracting energy-consuming chromatin remodelers. This subset includes genes that initiate EBV's lytic phase and are essential for lytic viral DNA replication (*BMLF1*, *BRLF1*, *BMRF1*). Other early lytic promoters underwent sufficient changes of the nucleosome occupancy also when BZLF1's transactivation domain was missing. The truncated BZLF1 protein has an additional C-terminal domain of debated function, which could also contribute to the recruitment of remodeling factors [41]–[44]. In the moment it is uncertain whether BZLF1's DNA binding domain alone is sufficient to evict nucleosomes.

BZLF1 provides a mechanism bypassing epigenetic repression and governing chromatin transitions in EBV. Our data indicate that the rapid reversal of Polycomb silencing, nucleosome remodeling, establishment of the activation mark H3K4me3, binding of PolII, and transcriptional initiation are controlled temporally. The activation of EBV's early lytic promoters first requires a loss of repressive modifications, before PolII is able to bind to and activate the promoter regions. The activation mark H3K4me3 is established upon polymerase binding, labeling actively transcribed genes. Surprisingly, these chromatin transitions occur in the presence of CpGs that maintain their fully methylated state throughout lytic gene expression.

Very much in contrast to EBV, KSHV relies on bivalent chromatin for maintaining its latent state, which is poised for transcriptional activation and readily supports escape from latency [34], [35]. It seems that KSHV counts on bivalent chromatin as one option of viral reactivation because it lacks a functional analogue of *BZLF1*. KSHV encodes a *BZLF1* homologue, which does not share the characteristics of EBV's BZLF1 to bind to CpG-methylated DNA sequence motifs (unpublished data). Bivalent chromatin is advantageous when it comes to reactivating from latency but it also comes at a cost because it will occasionally and stochastically trigger low-level viral gene expression, which will not go unnoticed by the immune system of the KSHV-infected host, eradicating the virus-infected cell.

In contrast, EBV adopts a fully silenced state of gene expression in memory B cells *in vivo* [45], [46], hiding from the host's educated immune system. Our findings provide a molecular explanation why EBV can afford to adopt an almost perfect state of viral latency during which all but two viral genes, *EBER1* and *-2*, are silenced. In this situation, which is also termed latency 0 [47], the exceptional characteristics of BZLF1 alone can reactivate the fully repressed viral chromatin to enable escape from latency. Upon B-cell receptor-mediated activation, BZLF1 is expressed in the latently infected cell capable of binding to meZRE sites [32]. We show in this report that it acts as a pioneer factor reverting repressed, inactive, and completely CpG-methylated viral chromatin. It is interesting to note that CpG dinucleotides in the *BZLF1* promoter are unusually rare and unmethylated in Raji cells but also in memory B cells *in vivo*. Our findings also indicate that the meZRE class of binding sites are not involved in the regulation of *BZLF1* itself, which relies on positive feedback loops [48], [49]. Other mechanisms control and repress the *BZLF1* promoter during latency [50 and references therein] and presumably contribute to its reactivation. In essence, EBV's hiding silently during latency is beneficial for a stable and successful virus-host relationship and seems to be superior to KSHV's strategy relying on bivalent chromatin, which might suffer from occasional leakage. It is well known that KSHV is less prevalent in the human population presumably because in an immunocompetent host, it gets eradicated whereas EBV does not. In the immunocompromised patient, KSHV can give rise to B cell lymphomas as well as to Castleman's disease and Kaposi's sarcoma, which all express latent genes together with a subset of lytic viral genes, very much in contrast to most EBV-associated malignancies, in which only latent genes are expressed.

The epigenetic regulation of the viral life cycle is the key to EBV's success infecting and persisting in its host, but might also apply to cellular promoters as a response to stress, e.g. UV-irradiation or inflammatory cytokines. It seems unlikely that BZLF1 is the only pioneer factor that can overcome transcriptional repression of highly CpG-methylated genes. Cellular factors of the AP-1 family to which BZLF1 belongs are likely candidates as they can reactivate repressed genes in response to exogenous signals in quiescent cells similar to BZLF1.

## Materials and Methods

### Purification of memory B cells

PBMCs were prepared from buffy coats of anonymous donors (Institute for Transfusion Medicine, Ulm, Germany) and purified by multi-parameter cell sorting on a FACS Aria III instrument (Becton-Dickinson). Cells were stained with anti-human CD19 (coupled to eFluor 450; eBioscience), anti-human CD38 (clone HIT2, coupled to PE; eBioscience), anti-human IgD (coupled to FITC; BD Pharmingen) and anti-human CD27 (APC conjugated; BD Bioscience). The stained cells were sorted for CD19 positive, CD38 and IgD negative, and CD27 positive cells as described previously [18].

### Plasmids

The plasmid pRTS-2 comprises the constitutively active, bicistronic coding sequence of the Tet-repressor-KRAB fusion gene and the tetracycline controlled transactivator rtTA2s-M2 [51]. The coding sequences of *BZLF1* and the green fluorescent protein (GFP) were placed under the control of the *tet* promoter to obtain an inducible *BZLF1* expression that can be monitored by GFP positive cells with, for example, flow cytometry.

### Deep bisulfite sequencing

Bisulfite modification of DNA was carried out using the EZ Methylation Gold Kit (Zymo). 100 nanogram each of 60 PCR products of bisulfite-modified DNA with an average product size of 463 bps were pooled for deep sequencing on a Roche Genome Sequencer FLX (Microsynth GmbH, Switzerland). Alignment of sequences was done using the CLC genomics workbench software with parameters for high throughput sequencing in the menu “reference assembly”. The local alignment was performed with parameters for long run reads (mismatch cost = 2, insertion cost = 3, deletion cost = 3, length fraction = 0.5 and similarity of 0.9). Sequence reads were aligned to a modified version of the B95.8 wildtype sequence, in which we converted all cytosines in a non-CpG context to thymines. Cytosines of CpG dinucleotides were kept unconverted. The parameters of the sequence alignment were set to tolerate RY-mismatches in the context of CpG dinucleotides, allowing mismatches of purines and pyrimidines, which are expected if cytosines are unmethylated. Non-specific matches were placed randomly. Methylation analysis was carried out using the BiQ analyzer HT 0.9/beta-test version [52]. Data were graphically analyzed and visualized with the Prism software package.

### MND-on-Chip analysis

1×10^7^ cells were harvested, washed once with PBS, and resuspended in 250 µl permeabilizing buffer (150 mM sucrose, 50 mM Tris-HCl, pH 7.9, 50 mM NaCl, 2 mM CaCl_2_). Cells were incubated for two minutes at 37°C in the presence 0.1% lysolecithine. MNase digestion was carried out using 50 U MNase for five minutes at 37°C. After MNase treatment, cells were lysed in TNESK buffer (20 mM Tris-HCl, pH 7.9, 200 mM NaCl, 2 mM EDTA, 2% SDS, 20 µg/ml Proteinase K) and incubated at 50°C for five hours. DNA was phenol/chloroform purified, precipitated with ethanol and sodium acetate, and loaded quantitatively on an agarose gel to purify the mononucleosomal DNA. Gel purification was carried out using the NucleoSpin Extract II Kit (Macherey-Nagel). For the preparation of input DNA, DNA was extracted from whole cells using the QIAamp DNA Mini Kit. DNA was sheared 15 times for five minutes in a biorupter device (Diagenode) to obtain DNA fragment sizes similar to MNase digested DNA. Mononucleosomal DNA and sonicated input DNA with an average length of 150 bp were labeled with the fluorochromes Cy5 and Cy3, respectively, and hybridized to a high-resolution EBV microarray. The microarray contained the whole EBV B95.8 sequence in tiling oligonucleotides of 50 nucleotides in a step size of ten and an offset of five nucleotides between upper and lower DNA strand to provide a final resolution of five nucleotides. DNA was chemically labeled with the Universal Linkage System (ULS) of Kreatech diagnostic. Labeling and hybridization was done in cooperation with Imagenes according to their standard protocols.

### Bioinformatical evaluation of nucleosome occupancies at BZLF1-regulated promoters

Raw data from the custom Nimblegen microarrays (.PAIR format) were analyzed with the software “R” (http://www.r-project.org) and the software “Bioconductor” (http://www.bioconductor.org). All functions were called using default parameters if not indicated otherwise. Raw data signals of all replicates were “scale normalized” to compensate for potential biases introduced during the manufacturing process [53]. The log_2_-ratios (enriched/input) of the normalized signals were determined and averaged for each replicate. The ZREs that had been identified in the EBV genome [32] were reviewed for the following criteria for a subsequent bioinformatical analysis: Clusters of ZREs and meZREs or single BZLF1 binding sites had to be at least 1500 bp apart from each other in the EBV genome. In regions that encompass two or more closely spaced ZREs in a cluster, the position with the strongest binding of BZLF1 was selected and included in the analysis. 32 ZREs and meZREs fulfilled the criteria and were selected for the analysis (see Table S2) Average nucleosome occupancies were determined separately for the three different conditions: Raji cells, Raji-BZLF1ΔTAD cells, and lytically induced Raji-BZLF1 cells. A window of ±2000 bp, centered at the start position of each ZRE, was chosen. The log_2_-ratios (enriched/input) were averaged in sliding sub-windows of 150 bp, which is approximately the length of one nucleosome, and a step size of 10 bp. To identify differences between the latent and lytic phase, subtractions of log_2_-ratios (enriched/input) of lytically induced Raji-BZLF1 cells and parental Raji cells were calculated for each individual ZRE and visualized in a heat map. A hierarchical cluster analysis using the Ward's minimum variance method was performed on the same heat map matrix with a window of ±350 bp, centered at the start position of each ZRE to probe for functional groups among ZREs. Average nucleosome occupancy profiles of each group identified by the cluster analysis were presented separately. As a second step, subtraction of log_2_-ratios (enriched/input) and cluster analysis was performed on ZREs that comprised a difference between the latent and lytic states with the datasets Raji-BZLF1 and Raji-BZLF1ΔTAD to determine the role of BZLF1's activation domain on nucleosome occupancies. 32 randomly chosen positions that lack ZREs were chosen and analyzed similar to the ZREs as a control.

### Chromatin Immunoprecipitation (ChIP)

ChIP experiments were conducted following standard protocols. Chromatin was cross-linked for seven minutes at room temperature and incubated with anti-H3 (Abcam, #1791-100), anti-H3K4me3 (Active Motif, #39159), anti-H3K27me3 (Upstate, #17-622), anti-RNA polymerase II (N20, Santa Cruz, #39097), anti-EZH2 (Active Motif, #39875) and anti-H3K9me3 (Active Motif #39161) antibodies. Protocol details are available upon request. ChIP and input samples were analyzed by quantitative real time PCR with the Roche LightCycler 480. Primer pair sequences are available on request. P values were determined using a non-paired homoscedastic t-test.

### Indirect endlabeling

MNase digestion was performed similarly to the sample preparation for microarray hybridization but with minor changes. Buffer volumes were increased to 1 ml to ensure proper isolation of DNA after MNase treatment and MNase digestion was performed with 0.1–1.5 U MNase. Chromatin was Proteinase K treated and purified by phenol/chloroform extraction and ethanol precipitation. Cleavage of MNase treated DNA was carried out using 30 µg of DNA and 100 U of the specific restriction endonuclease at 37°C for two hours. Samples were purified and analyzed in Southern blot hybridizations according to standard protocols.

### Western blot immunodetection

After polyacrylamid gel electrophoresis and membrane transfer an anti-EZH2 antibody (Active Motif, #39933) or an anti-α-tubulin antibody (Santa Cruz, SC-23948) were used for protein detection.

### RT-PCR analysis

Isolation of RNA from 1×10^7^ cells per sample was carried out with the RNase Mini Kit (Qiagen). The lysate was homogenized with QiaShredder columns (Qiagen). All subsequent steps were performed according to the instructions of the manufacturer. RNA was eluted in 80 µl of RNase-free water.

Prior to cDNA synthesis, contaminating DNA was removed from the RNA preparation with the enzyme DNase I (Invitrogen). 2 µg of extracted RNA were incubated for 90 min at 37°C in the presence of 2 U DNase, 40 U RNase inhibitor and 1× DNase buffer in a 20 µl approach. Remaining DNase was heat inactivated by incubating at 65°C for ten minutes. The efficiency of DNase treatment was controlled by PCR. Reverse transcription of RNA was performed with the SuperScript III First Strand Synthesis SuperMix Kit (Invitrogen) according to the manufacturer's instructions. Quantification of cDNA was done with quantitative real time PCR using the Roche LightCycler 480 system. Primer sequences are available on request.

### Intracellular staining of the R antigen by flow cytometry

Cells were harvested, washed with PBS and fixed with 1% paraformaldehyd for 15 min at room temperature. Fixed cells were washed with PBS +2% FCS and permeabilized with 80% methanol for 30 min at −20°C. The cells were washed with PBS +2% FCS and incubated with a 1∶20 dilution of a BRLF1 antibody (8C12) [54] for 30 min at room temperature. Cells were washed five times with PBS +2% FCS and subsequently stained with a 1∶5 dilution of an APC-coupled goat-anti mouse IgG antibody (Biolegend) for 30 min at 4°C. Cells were washed and analyzed by flow cytometry.

## Supporting Information

Figure S1 **Graphical representation of selected regions of the viral genome in Raji cells with results from deep bisulfite sequencing.** (**A**) Overview of the EBV genome with selected features and regions analyzed by bisulfite sequencing indicated in red. (**B**) Selected results of the deep sequencing analysis. Each bar represents a CpG dinucleotide and is plotted versus its genome coordinate on the x-axis. The percentage of methylation is displayed on the left y-axis and is indicated in red. The right y-axis provides the sequencing coverage, which is depicted as a grey area in the diagrams in logarithmic scale. Coverage above ten reads/bp was considered to be representative for the analysis. Deep bisulfite sequencing detected single sequence variations between Raji DNA and B95.8 wildtype sequence. CpG dinucleotides missing in the Raji genome but present in B95.8 are indicated as black bars with a star on top, while additional CpG dinucleotides in Raji cells are indicated with a circle on top of the red or grey bar. Selected annotation of the EBV genome can be found above the graph, with ZREs and meZREs indicated as black or red circles, respectively. Shown here are examples for latent gene promoters and region (*Cp*, *Wp*, *LMP2a*, and *LMP1* promoter), for BZLF1-regulated gene promoters (*BHLF1/BHRF1* promoter, *BFLF1/BFRF1* promoter, *BMRF1* promoter, *BMLF1* promoter, *BSRF1* promoter, *BRLF1* promoter, *BBLF2*/3 promoter and *BALF5* promoter) and lytic gene promoters (*BORF1* promoter, *BMRF2* promoter, *BLLF1* promoter, *BGRF1* promoter, *BDLF4/BDRF1* promoter and BcLF1 promoter). The latent Cp and Wp are heavily methylated in Raji cells. The latent LMP1 promoter is only slightly methylated and probably in an open configuration during latency. The promoters of *BMRF1*, *BMLF1*, *BSRF1*, *BRLF1*, *BBLF2/3*, and *BALF5* are examples for promoters that are bound by BZLF1 in a methylation dependent manner. The majority of CpG dinucleotides in the promoter regions appear hypermethylated. Only *BMRF1* shows some hypomethylated CpG dinucleotides. But all CpG dinucleotides in meZREs are methylated to nearly 100%. The promoters of *BHLF1/BHRF1* and *BFLF1/BFRF1* carry ZREs without a CpG site. The first one is variably methylated, while the latter is highly methylated in latent Raji cells. The lytic promoters, that do not carry binding sites for BZLF1 show an overall high degree of methylation, with some CpG sites that are unmethylated, for example in the BGRF1 promoter. Whether these sites are important for their activation needs to be addressed.
(PDF)

Figure S2 **Conditional expression of** ***BZLF1*** **in Raji cells.** (**A**) Schematic drawing of the plasmid 3862 pRTS-2-BZLF1. The plasmid contains the bicistronic coding sequence of the Tet-repressor-KRAB fusion gene and the tetracycline controlled transactivator rtTA2s-M2 under the control of the constitutively active chicken beta-actin promoter. The two genes are separated by an internal ribosomal entry site (IRES). In absence of tetracycline or its analogue, doxycycline, the repressor binds tightly to the so-called *tet* promoter and inhibits its activation. After addition of doxycycline to the cells, the repressor is released from the promoter and replaced by the activator, which induces transcription. The promoter in the plasmid is bidirectional, allowing the simultaneous expression of the two transgenes *GFP* and *BZLF1*. (**B**) GFP expression in single Raji cells is a measure of induced transgene expression of *BZLF1*. The addition of 100 ng/ml doxycycline to the cells for 15 hours led to 87% of GFP positive cells, as measured by flow cytometry. (**C**) BZLF1 expression can be monitored by quantitative RT-PCR. The amount of transcripts per cell rises quickly after treatment of Raji 3862 cells with doxycycline, peaking at eight hours post induction. (**D**) Intracellular staining for the BRLF1 antigen shows the induction of BZLF1 target genes in lytically induced cells. Top panel: B95.8 cells show a fraction of about 4% BRLF1-positive cells indicating spontaneous lytic induction. Addition of doxycycline did not increase the percentage of BRLF-positive cells, as expected. Middle panel: 75% of doxycycline-treated 3862 cells were GFP-positive after doxycycline induction in this experiment. Bottom panel: The same cells were fixed with paraformaldehyd and permeabilized with methanol and stained for intracellular BRLF1 protein. 56% of GFP-positive cells expressed BRLF1 detectably in this experiment. The reduction of GFP-positive cells as compared to the analysis shown in the middle panel can be explained by a loss of GFP protein and a decrease of GFP fluorescence during permeabilizing and fixating of the cells, respectively.
(EPS)

Figure S3 **Nucleosome occupancy in 32 randomly chosen positions that lack ZREs.** 32 randomly chosen positions without ZREs were bioinformatically evaluated similar to Fig. 2. Left panel: Subtractions of the log_2_-ratios of lytically induced Raji-BZLF1 cells and latent parental Raji cells for the 32 positions were visualized in a heat map. The subtraction of the two datasets mostly resulted in values close to zero, indicating that there was no significant difference between the datasets. The dendrogram clustered the positions into three groups, but the datasets did not display a noticeable difference as compared to Fig. 2. Right panel: Average nucleosome occupancy profiles of parental Raji cells (black line), Raji-BZLF1ΔTAD cells (green line) and lytically induced Raji-BZLF1 cells (red line) of the 32 positions did not reveal obvious differences between the analyzed datasets of three different Raji derivates.
(EPS)

Figure S4 **Close-up of six single ZREs of different ZRE groups.** MND-on-Chip experiments in conjunction with bioinformatical analysis identified ZREs, which belong to different groups. The nucleosome occupancy profiles of two members of each group are shown in a window of ±1000 bp, centered at the start of the ZRE. (**A**) Nucleosome occupancy profiles of two members of subgroup 2b. Subgroup 2b of ZREs had a high average nucleosome occupancy during latency (parental Raji cells, black line), which dramatically dropped after induction of the lytic phase (induced Raji-BZLF1 cells, red line). This observation was confirmed by looking at the individual nucleosome occupancy profiles of the promoters of *BMLF1* and *BRLF1*. The binding of the DBD of BZLF1, only, (Raji-BZLF1ΔTAD cells, green line) induced modest changes at the ZRE of the *BRLF1* promoter, but did not modify the nucleosome occupancy at the *BMLF1* promoter. (**B**) Nucleosome occupancy profiles of two members of subgroup 2a. ZREs of the subgroup 2a displayed high average nucleosome occupancy in latency in parental Raji cells, similar to subgroup 2b. Unlike ZREs of the subgroup 2b, BZLF1 DBD alone was sufficient to rearrange and diminish the average nucleosome occupancy (green line). Full length BZLF1 did not alter the average nucleosomal pattern any further (red line). The *BBLF4* promoter and the *BKRF3* promoter exemplify the nucleosome pattern of members of this subgroup. (**C**) Nucleosome occupancy profiles of two members of group 1. ZREs of group 1 comprised moderate average nucleosome occupancy during latency. Upon induction of the lytic phase, this nucleosomal pattern remained stable, as it was observed in the *BARF1* promoter and the ZRE in the *BNRF1* reading frame.
(EPS)

Figure S5 **ChIP-Bisulfite Sequencing (ChIP-BS-seq.) of the** ***BBLF4*** **promoter.** Raji-BZLF1 cells were induced with doxycycline for 15 h. A native chromatin-immunoprecipitation (ChIP) with a BZLF1 antibody (BZ1) was performed to enrich for DNA bound by BZLF1. The immunoprecipitated DNA was subsequently modified with bisulfite, amplified with primers covering four meZREs of the *BBLF4* promoter, and sequenced by conventional Sanger sequencing. (**A**) BZLF1 target sites were successfully enriched by immunoprecipitation. Immunoprecipitated DNA was analyzed by quantitative real-time PCR to assess the enrichment of the BZLF1 target promoter *BBLF4* relative to the reference locus *GAPDH*, which is not bound by BZLF1. Induced Raji-BZLF1 cells showed a 72.6 fold enrichment as compared to the uninduced control. (**B**) The methylation profile of the *BBLF4* promoter after BZLF1-specific ChIP of induced and uninduced cells is nearly identical, indicating that BZLF1 preferentially binds to methylated DNA and does not select non-methylated EBV DNA.
(EPS)

Table S1 **Methylation state of analyzed meZREs.**
(PDF)

Table S2 **List of ZREs that were part of the MND-on-Chip analysis.**
(PDF)
