# Supplementary figures and images for: BZLF1 Governs CpG-Methylated Chromatin of Epstein-Barr Virus Reversing Epigenetic Repression
Source: PLoS Pathog. 2012 Sep 6;8(9):e1002902. doi: 10.1371/journal.ppat.1002902 (PMC3435241; doi:10.1371/journal.ppat.1002902)

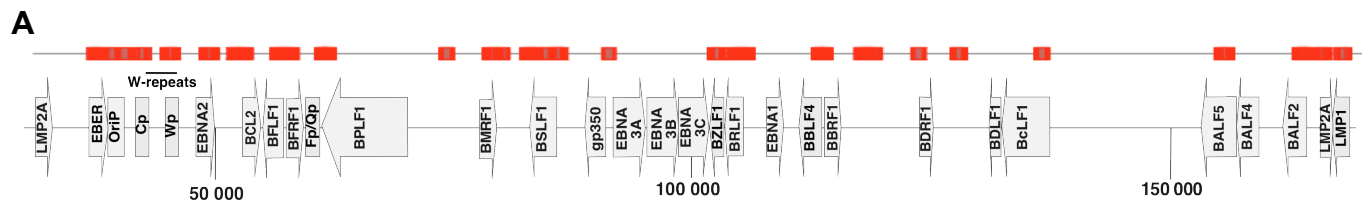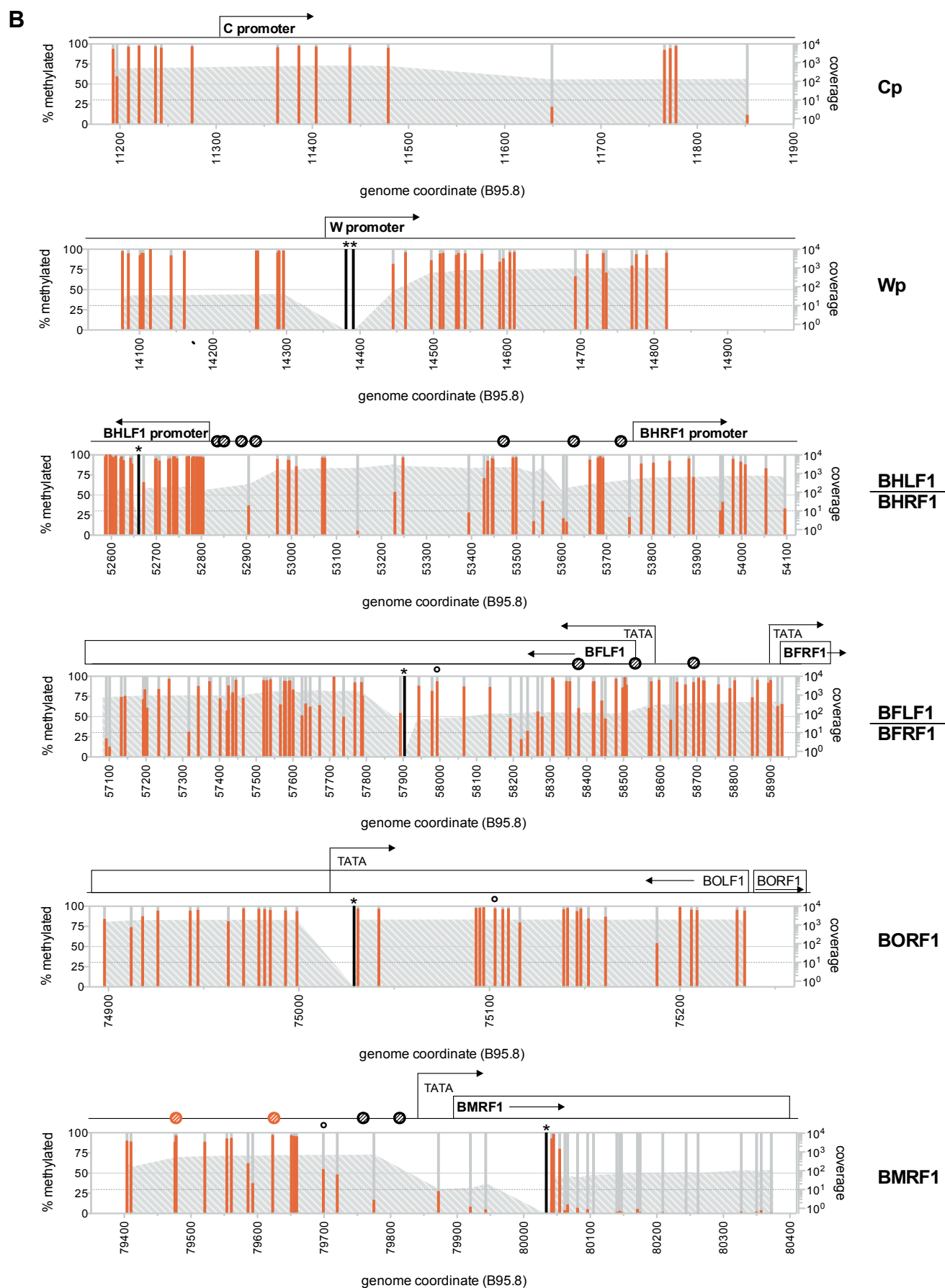

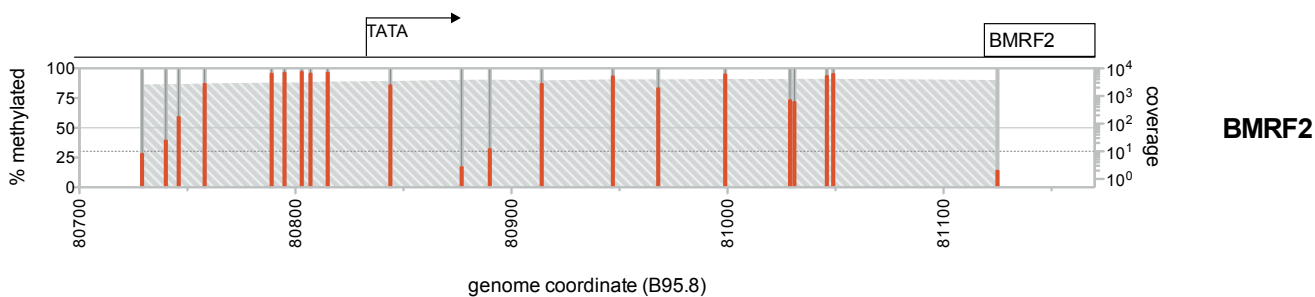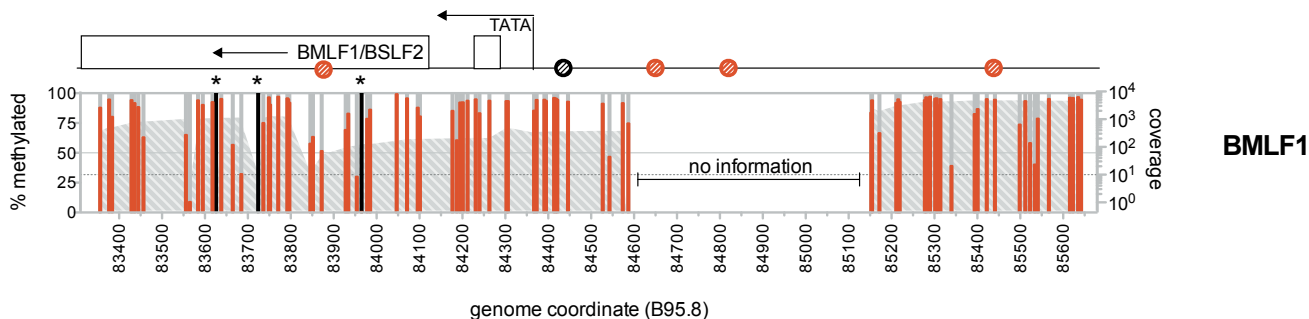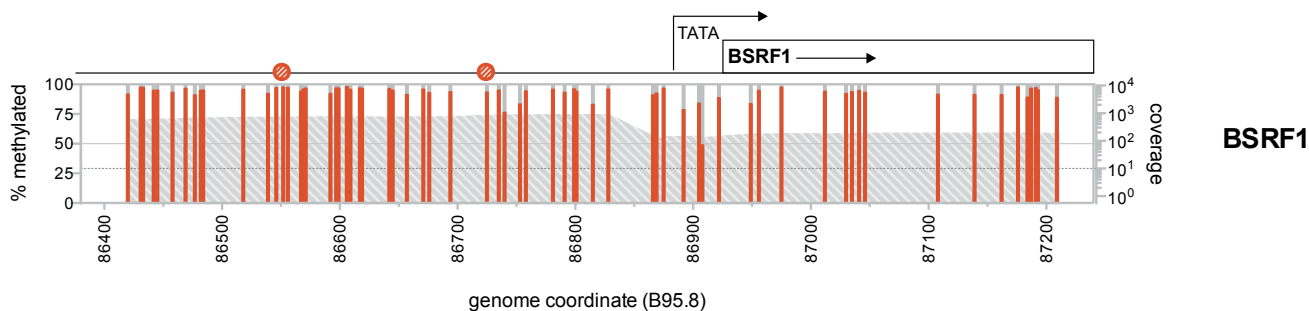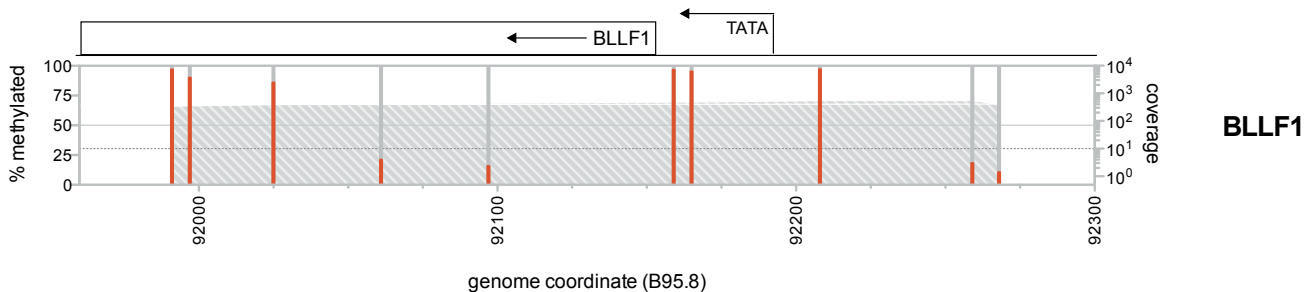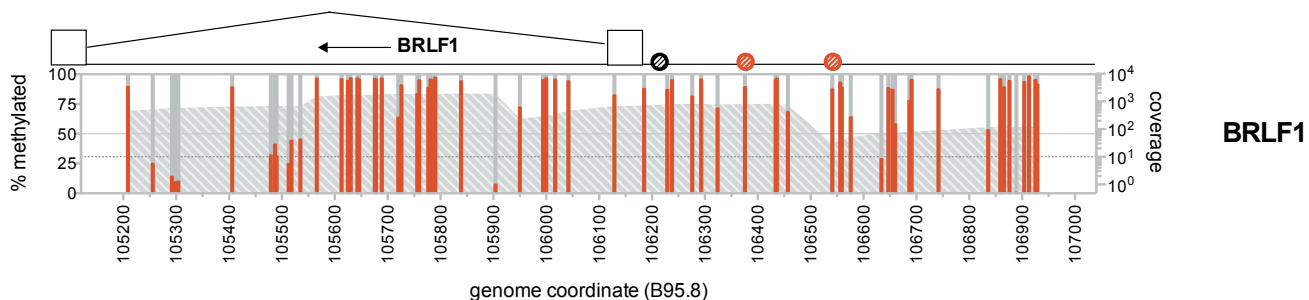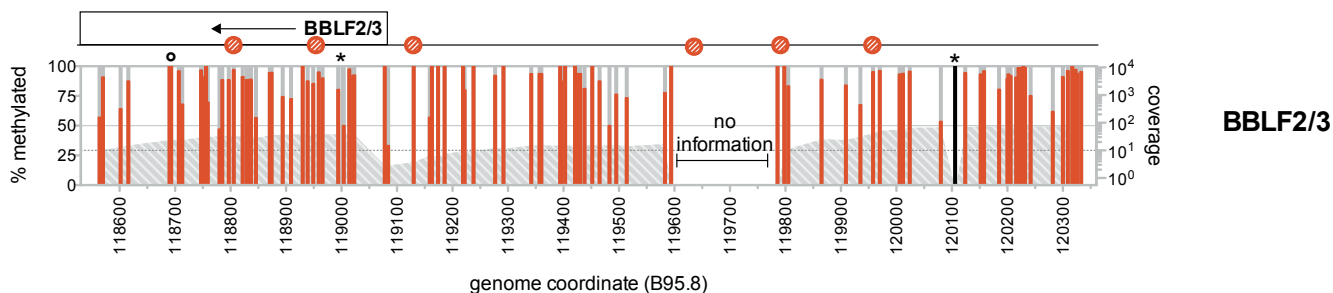

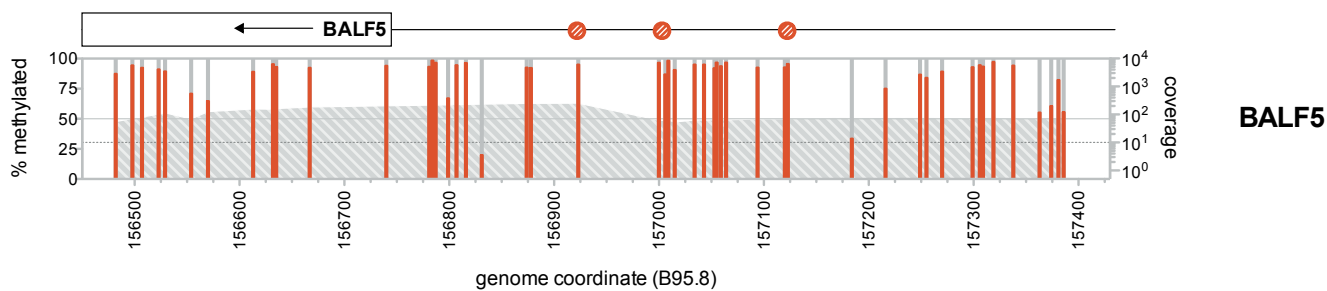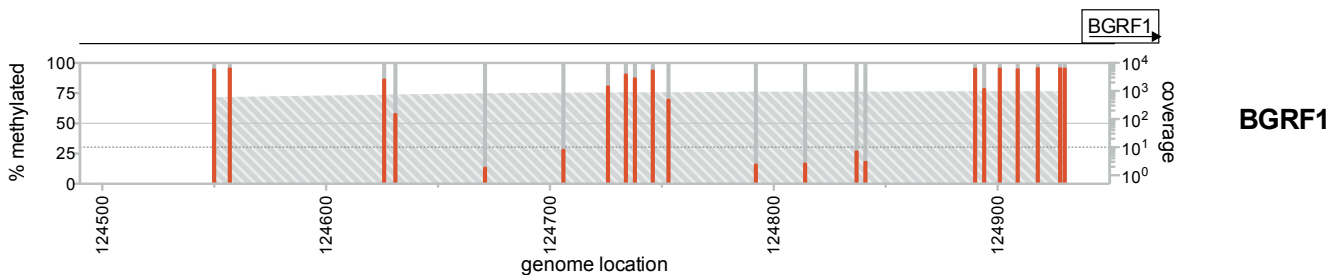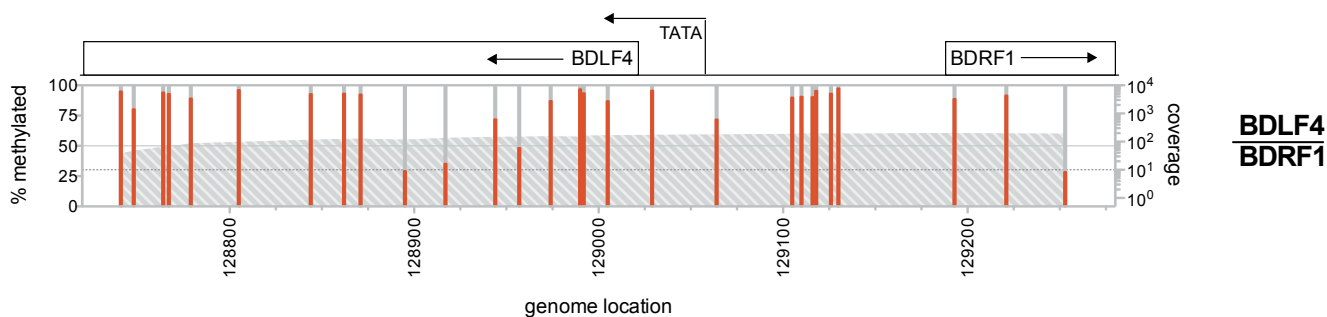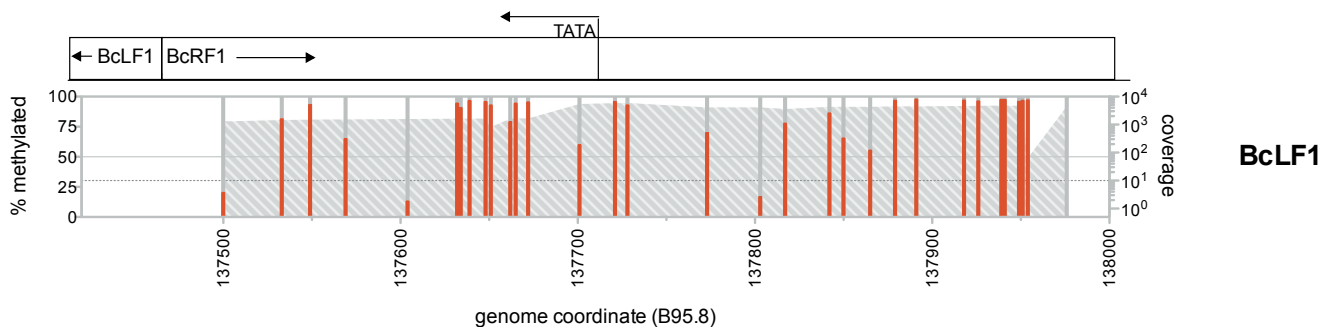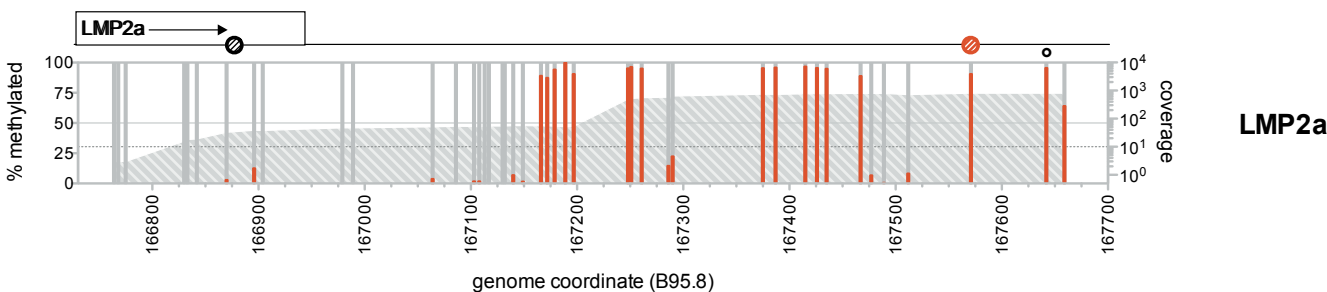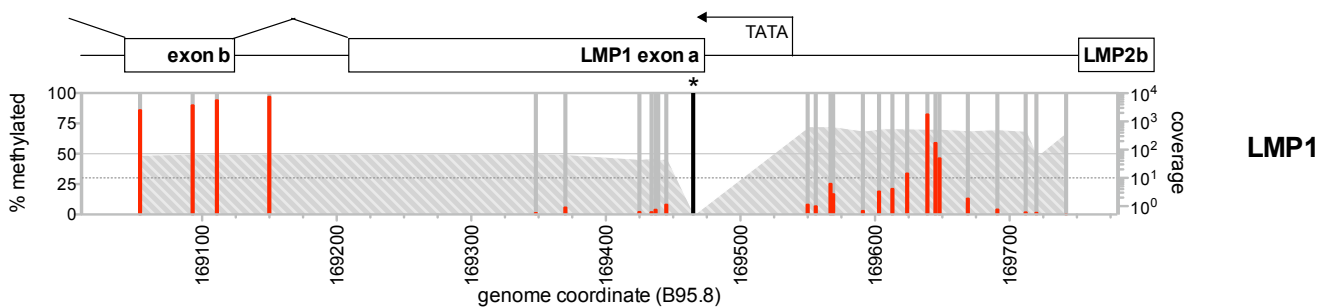

Supplement: Figure S1 — Graphical representation of selected regions of the viral genome in Raji cells with results from deep bisulfite sequencing. (A) Overview of the EBV genome with selected features and regions analyzed by bisulfite sequencing indicated in red. (B) Selected results of the deep sequencing analysis. Each bar represents a CpG dinucleotide and is plotted versus its genome coordinate on the x-axis. The percentage of methylation is displayed on the left y-axis and is indicated in red. The right y-axis provides the sequencing coverage, which is depicted as a grey area in the diagrams in logarithmic scale. Coverage above ten reads/bp was considered to be representative for the analysis. Deep bisulfite sequencing detected single sequence variations between Raji DNA and B95.8 wildtype sequence. CpG dinucleotides missing in the Raji genome but present in B95.8 are indicated as black bars with a star on top, while additional CpG dinucleotides in Raji cells are indicated with a circle on top of the red or grey bar. Selected annotation of the EBV genome can be found above the graph, with ZREs and meZREs indicated as black or red circles, respectively. Shown here are examples for latent gene promoters and region (Cp, Wp, LMP2a, and LMP1 promoter), for BZLF1-regulated gene promoters (BHLF1/BHRF1 promoter, BFLF1/BFRF1 promoter, BMRF1 promoter, BMLF1 promoter, BSRF1 promoter, BRLF1 promoter, BBLF2/3 promoter and BALF5 promoter) and lytic gene promoters (BORF1 promoter, BMRF2 promoter, BLLF1 promoter, BGRF1 promoter, BDLF4/BDRF1 promoter and BcLF1 promoter). The latent Cp and Wp are heavily methylated in Raji cells. The latent LMP1 promoter is only slightly methylated and probably in an open configuration during latency. The promoters of BMRF1, BMLF1, BSRF1, BRLF1, BBLF2/3, and BALF5 are examples for promoters that are bound by BZLF1 in a methylation dependent manner. The majority of CpG dinucleotides in the promoter regions appear hypermethylated. Only BMRF1 shows some hypomethylated CpG [file ppat.1002902.s001.pdf]
