# Supplementary material for: BZLF1 Governs CpG-Methylated Chromatin of Epstein-Barr Virus Reversing Epigenetic Repression
Source: PLoS Pathog. 2012 Sep 6;8(9):e1002902. doi: 10.1371/journal.ppat.1002902 (PMC3435241; doi:10.1371/journal.ppat.1002902)
Supplement: Table S1 — Methylation state of analyzed meZREs. (PDF) [file ppat.1002902.s006.pdf]

Table S1 Methylation state of analyzed meZREs.

meZRE	methylation state (%)
79478	96.08
79623	96.74
83872	51.67
85441	94.75
86552	97.76
86725	93.39
106376	89.36
106541	87.50
114354	95.45
114431	89.12
114499	97.37
114621	81.20
114805	91.48
118806	97.06
118949	85.37
119130	100.00
119798	100.00
119958	95.45
156923	95.31
157000	96.77
157120	92.86
