# Supplementary material for: BZLF1 Governs CpG-Methylated Chromatin of Epstein-Barr Virus Reversing Epigenetic Repression
Source: PLoS Pathog. 2012 Sep 6;8(9):e1002902. doi: 10.1371/journal.ppat.1002902 (PMC3435241; doi:10.1371/journal.ppat.1002902)
Supplement: Table S2 — List of ZREs that were part of the MND-on-Chip analysis. (PDF) [file ppat.1002902.s007.pdf]

Table S2 List of ZREs that were part of the MND-on-Chip analysis.

ID	ZRE type	start of ZRE	end of ZRE	EBV genome feature
1	meZRE	2531	2538	BNRF1 reading frame
2	meZRE	4275	4282	BNRF1 reading frame
3	ZRE	9159	9166	BCRF1 reading frame
4	ZRE	49288	49295	n.o.s.*
10	ZRE	53630	53636	oriLyt
12	meZRE	56777	56784	BFLF2 promoter
14	ZRE	58518	58524	BFLF1 promoter
16	meZRE	64602	64609	BPLF1 reading frame
19	meZRE	77941	77948	Barf1 promoter
21	ZRE	79760	79766	BMRF1 promoter
24	meZRE	84646	84653	BMLF1 promoter
26	meZRE	86548	86555	BSRF1 promoter
28	meZRE	91000	91007	BLLF1 reading frame
29	ZRE	93977	93984	n.o.s.*
30	ZRE	97961	97968	n.o.s.*
31	ZRE	103303	103311	BZLF1 promoter
34	meZRE	106372	106378	BRLF1 promoter
36	ZRE	110108	110115	BKRF3 promoter
38	meZRE	114495	114502	BBLF4 promoter
40	meZRE	119796	119803	BBLF2 promoter
41	ZRE	122476	122483	BGLF5 promoter
42	meZRE	126796	126803	BGLF2 promoter
44	ZRE	129593	129600	BDLF4 promoter
45	ZRE	131379	131386	BDLF3 promoter
47	ZRE	137155	137162	n.o.s.*
48	ZRE	145154	145131	BVRF1 promoter
51	ZRE	148198	148205	BdRF1 promoter
52	ZRE	150517	150524	BILF2 promoter
54	ZRE	152264	152271	n.o.s.*
55	meZRE	155182	155189	BALF5 reading frame
56	meZRE	156993	157000	BALF5 promoter
59	meZRE	167567	167574	BNLF2a/b promoter

*n.o.s. not otherwise specified
